# Bioavailability of Dietary Polyphenols and Gut Microbiota Metabolism: Antimicrobial Properties

**DOI:** 10.1155/2015/905215

**Published:** 2015-02-23

**Authors:** Laura Marín, Elisa M. Miguélez, Claudio J. Villar, Felipe Lombó

**Affiliations:** Research Unit “Biotechnology and Experimental Therapy Based in Nutraceuticals-BITTEN”, Instituto Universitario de Oncología del Principado de Asturias (IUOPA), Universidad de Oviedo, 33006 Oviedo, Spain

## Abstract

Polyphenolic compounds are plant nutraceuticals showing a huge structural diversity, including chlorogenic acids, hydrolyzable tannins, and flavonoids (flavonols, flavanones, flavan-3-ols, anthocyanidins, isoflavones, and flavones). Most of them occur as glycosylated derivatives in plants and foods. In order to become bioactive at human body, these polyphenols must undergo diverse intestinal transformations, due to the action of digestive enzymes, but also by the action of microbiota metabolism. After elimination of sugar tailoring (generating the corresponding aglycons) and diverse hydroxyl moieties, as well as further backbone reorganizations, the final absorbed compounds enter the portal vein circulation towards liver (where other enzymatic transformations take place) and from there to other organs, including behind the digestive tract or via blood towards urine excretion. During this transit along diverse tissues and organs, they are able to carry out strong antiviral, antibacterial, and antiparasitic activities. This paper revises and discusses these antimicrobial activities of dietary polyphenols and their relevance for human health, shedding light on the importance of polyphenols structure recognition by specific enzymes produced by intestinal microbial taxa.

## 1. Bioavailability of Dietary Polyphenols

### 1.1. Structural Diversity

Flavonoids are very abundant 15C secondary metabolites in plants, containing two aromatic rings (connected by a heterocycle pyrone ring), which are tailored with diverse hydroxyl moieties. Some are produced at chloroplasts as defense against oxidative damage generated during photosynthesis [1]; others are produced at the sexual organs as defense against solar UV [2], at the root area as attractants for bacterial and fungal symbionts [3], or as defense against virus, bacteria, fungi, and herbivores [4].

**Figure 1:**
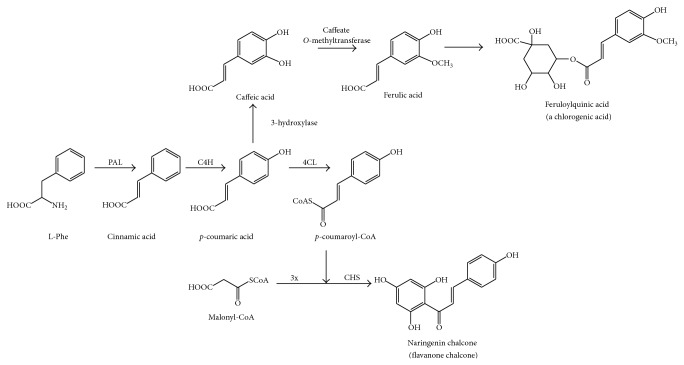
Initial common steps during hydroxycinnamic acids and flavonoids biosynthesis in plants.

All flavonoids derive from L-phenylalanine, due to diverse transformations taking place at the phenylpropanoid pathway. Initial common steps are conversion of L-Phe in cinnamic acid (by phenyl ammonia lyase (PAL)), its conversion in* p*-coumaric acid (by cinnamate-4-hydroxylase (C4H)), and its transformation in* p*-coumaroyl-CoA (by 4-coumaroyl-CoA ligase (4CL)) [5]. Both* p*-coumaric acid and* p*-coumaroyl-CoA are building blocks for hydroxycinnamic acids and flavonoids, respectively (Figure 1) [5].

**Figure 2:**
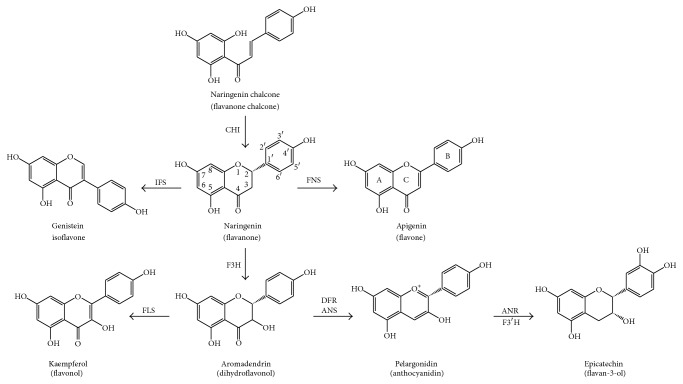
Biosynthetic steps for generation of flavonoid subfamilies. Naringenin structure shows atom numbering and apigenin structure shows rings denomination.

In flavonoid biosynthesis, one molecule of* p*-coumaroyl-CoA and three molecules of malonyl-CoA are used by the chalcone synthase (CHS) in order to generate a bicyclic chalcone as naringenin chalcone (Figure 1) [4]. Chalcones are substrates for chalcone isomerase (CHI), which carries out the B-ring closure at these compounds, rendering flavanones (as naringenin from citrus fruits) (Figure 2). All flavonoid subfamilies derive from these 15C flavanones (Figure 2 shows the atom numbering and ring denomination). Other phenylpropanoid enzymes will generate diverse final products as shown in Figure 2 [4]. Flavone synthase (FNS) will generate flavones (as apigenin from celery). Isoflavone synthase (IFS) will generate isoflavones (as genistein from soy). Flavanone-3-hydroxylase (F3H) will generate dihydroflavonols (as aromadendrin from pine trees). Flavonol synthase (FLS) will generate flavonols (as quercetin from onion or kaempferol from capers). Dihydroflavonol reductase (DFR) and anthocyanin synthase (ANS) will generate anthocyanidins (as pelargonidin from diverse red flowers). Anthocyanidin reductase (ANR) will generate flavan-3-ols (as epicatechin from cocoa).

Flavonoids are usually present and stored in plant tissues in the form of diverse derivatives, mostly as sugar O-conjugates at C2 (chalcones), at C3 (flavonols, anthocyanidins, and flavan-3-ols), or at C7 (flavanones, flavones, and isoflavones) positions. Most common bound sugars are glucose, galactose, rhamnose, xylose, rutinose, arabinopyranose, and arabinofuranose [6]. These modifications (and others as methylations and gallate tailoring) add extra structural stability to flavonoids during storage in vacuoles and chloroplasts [7–9]. Once the plant, fruit, or seed is recollected, flavonoids usually have good stability in this conjugated state and keep high concentrations in food and beverages. All these modifications in chemical structure and sugar binding will determine their absorption and bioavailability [10–13].

### 1.2. Intestinal Absorption

**Figure 3:**
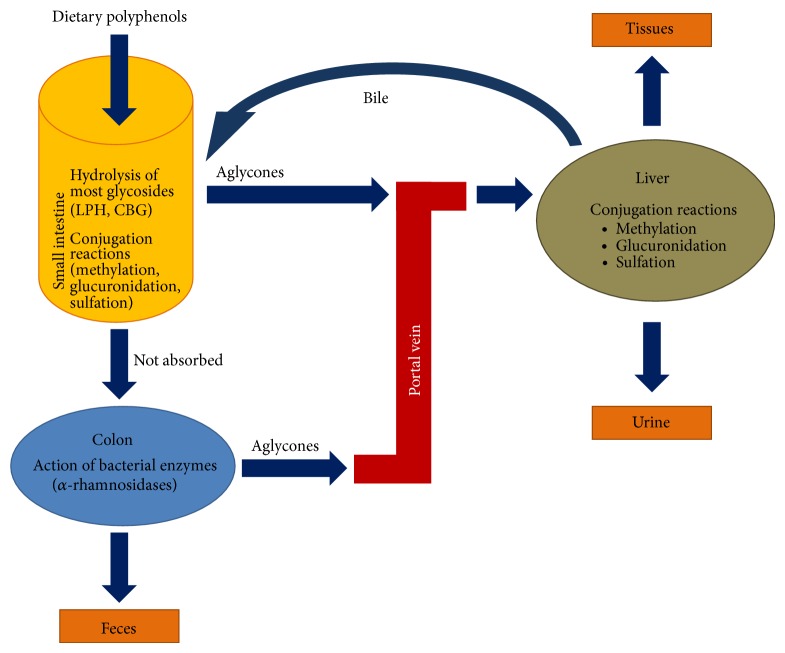
Absorption and metabolism routes for dietary polyphenols and their derivatives in humans.

The study of flavonoids metabolism in human body is crucial to determine which ones are better absorbed and which ones lead to formation of bioactive metabolites. Following the ingestion of flavonoids, sugar moieties (as in quercetin-3-glucoside) are cleaved from the phenolic backbone in the small intestine and absorbed here. Enzymes as lactase phlorizin hydrolase (LPH) (at enterocyte membrane) or *β*-glucosidase (CBG) (cytosolic, for polar glycosides) hydrolyze glycosylated flavonoids and then aglycones enter epithelial cells by passive diffusion [14–16] (Figure 3). However, flavonoids linked to a rhamnose moiety must reach the colon and be hydrolyzed by the *α*-rhamnosidases secreted by the colon microbiota (as* Bifidobacterium dentium*), in order to proceed to its absorption [17] (Figure 3). Flavan-3-ols, such as (−)-epicatechin, are never glycosylated but often acylated by gallic acid. These compounds are absorbed at enterocyte level without any deconjugation or hydrolysis [18]. Proanthocyanidins are polymers of high molecular weight, and therefore oligomers larger than trimers are unlikely to be absorbed in the small intestine in their native form [19].

**Figure 4:**
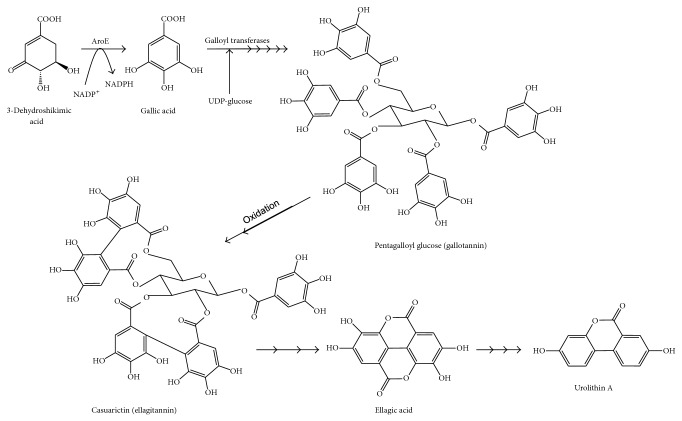
Biosynthetic steps for generation of two hydroxycinnamic acid polymers: ellagitannins and gallotannins.

The other main family of polyphenols, hydroxycinnamic acids, are commonly esterified to sugars, organic acids, and lipids. There are no esterases in human tissues able to break these ester links, so the main site for its metabolism is colonic microbiota, although up to one third of their absorption can also take place in the small intestine [46–49]. Some hydroxycinnamic acids, as ellagitannins, are polymers (Figure 4). These are resistant to the action of LPH or CBG and consequently cannot be absorbed in the small intestine, reaching the colon, where its microbiota cleaves the conjugating moieties. The resultant aglycones are extensively metabolized by this microbiota, leading to the production of various hydroxyphenylacetic acids [50, 51].

Once a final derivative or aglycon has been absorbed (at small intestine or colon), it undergoes some degree of phase II metabolism at enterocyte level, as methylation (at C3′ or C4′ by catechol-O-methyltransferase (COMT)), sulfation (at C3′, C4′, C5, or C7 by sulfotransferases (SULT)), and glucuronidation (by UDP-glucuronosyltransferases) (Figure 3). Then these products enter the blood stream by the portal vein, reaching the liver, where they may be subjected to more phase II metabolism, hence becoming conjugated and transported to the bloodstream again until they are secreted in urine (Figure 3) [25, 52–61]. Some of the liver conjugates are then excreted as bile components back into the intestine (enterohepatic recirculation) and deconjugated compounds are regenerated by gut microbial enzymes before being reabsorbed again [21, 62, 63]. The unabsorbed metabolites are eliminated via faeces (Figure 3). All these conjugation mechanisms are highly efficient, and free aglycones are generally absent or present in low concentrations in plasma after nutritional doses. An exception is green tea catechins, whose aglycones constitute a significant proportion of the total amount in plasma, as they are nonglycosylated flavonoids in food and are readily absorbed at the small intestine without extra modifications [12].

## 2. Antimicrobial Effects of Dietary Polyphenols and Their Gut Microbiota Metabolites

The level of biotransformations suffered by a specific dietary polyphenol along the gastrointestinal tract is determined by two main factors. One is the specific structural subfamily of the polyphenol, as its scaffold will allow only some transformations, to be carried out by intestinal enzymes and gut microbiota species. This chemical structure will therefore, at this initial level, restrict the range of possible final bioactive products to be absorbed and consequently the scale of possible antimicrobial properties generated as a result of these biotransformations on dietary polyphenols. The second factor is the individual richness at the level of intestinal microbiota, as some specific biotransformations on dietary polyphenols can be carried out by a vast array of gut microbial species and genera (as deglycosylations), but other more specific chemical reactions on polyphenols require the presence of particular species or strains gifted with special genes coding for precise enzymes (as those responsible for intestinal generation of urolithins or (*S*)-equol).

Along the next sections, intestinal transformations of dietary polyphenols by diverse microbiota species (and the antimicrobial bioactivities of those derivatives) are organized according to their different structural subfamilies.

### 2.1. Flavonols

Flavonols (kaempferol, quercetin, and myricetin) (Figure 2) share the 3-hydroxyflavone backbone. Different positions for the phenolic OH moieties give diversity to this subgroup. They are found as glycosylates in many common foods as onion, capers, apples, broccoli, grapefruit, and plums. One of the most important diet flavonols is quercetin, whose 4′-O-glucoside and 3,4′-O-diglucoside, among others, are abundant in onion and propolis, for example [64, 65].

The type of initial glycosylation pattern affects flavonols degradation rates in the gut. Metabolism of di- and trisaccharides is much slower compared to that of flavonol monosaccharides. Position of the hydroxyl groups may also influence their degradation, as recent studies indicate that flavonoids without hydroxyl groups at the C5, C7, and C4′ positions are degraded slower. Some gut microbiota species that have been involved in this hydrolysis are* Bacteroides distasonis*,* Bacteroides uniformis*,* Bacteroides ovatus*,* Enterococcus casseliflavus*, and* Eubacterium ramulus* [20, 21, 66, 67]. Also, the type of glycosidic bond (C- or O-glycosides) has influence on their degradation rates. Metabolism of a* C*-glycosidic bond seems to be much slower than the hydrolysis of an O-glycosidic bond. This is of interest from a nutraceutical point of view, as the slow degrading compounds may be more bioavailable, because they have greater opportunity to be absorbed than the ones that are degraded at a quicker rate at colon level [66].

**Figure 5:**
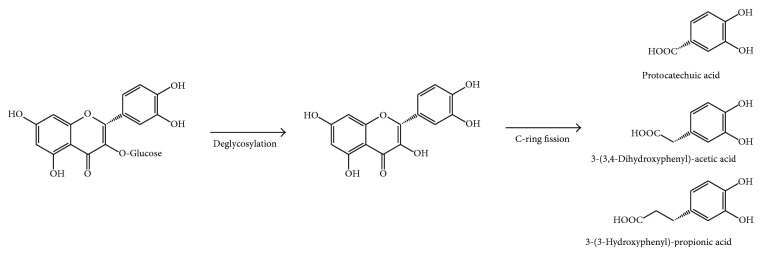
Colonic degradation of quercetin glycosides, as an example of flavonol glycosides.

Once flavonols have been metabolized in their aglycones, they are extensively degraded by other colonic microbiota, generating simpler phenolic compounds derived from A- and B-ring metabolism, after the flavonoid C-ring has been broken down [64] (Table 1, Figure 5). C-ring breakdown takes place at different positions (breaking the bond between C1 and C2 positions, between C3 and C4, or between C4 and C10) giving rise to a high number of simple phenolics (Table 1, Figure 5). Some gut microbiota involved in this C-ring breakdown is* Eubacterium oxidoreducens*,* E. ramulus*,* E. casseliflavus*,* Clostridium orbiscidens*, and others belonging to* Butyrivibrio* genus [20–23].

Following the C-ring fission, dehydroxylation occurs at the two remaining free phenolic rings (Figure 5). The hydroxylation pattern of A- and B-ring affects therefore the type of phenolic compounds produced, which will be finally absorbed at colon level. For example, the primary gut microbiota metabolites of quercetin are 2-(3,4-dihydroxyphenyl)-acetic acid (from A-ring) and protocatechuic acid (from B-ring), and those ones of myricetin are 2-(3,5-dihydroxyphenyl)-acetic acid (from A-ring) and gallic acid (from B-ring) (Figure 5). Further dehydroxylation results in the formation of 2-(3-hydroxyphenyl)-acetic acid from both metabolites [22, 23, 68].

With respect to bioactivity, flavonols have been described as antiviral, inhibiting HIV-1 integrase, although in a nonspecific way, but also against HSV, respiratory syncytial virus, and poliovirus [69, 70]. Quercetin has been shown to potentiate the action of acyclovir against HSV infection [71]. With respect to its antibacterial activity, oral administration of quercetin protected against* Shigella *infection in an animal model using a 140 mg/kg doses [72].* Escherichia coli *gyrase is inhibited by quercetin and other flavonols, by inhibiting the ATPase GyrB subunit [73]. In* in vitro *assays, quercetin increases the bacterial cell membrane, giving rise to dissipation in membrane potential, and diminished cell motility, which is an important factor in bacterial virulence [74].

### 2.2. Flavanones

This class of flavonoids (hesperetin, naringenin) (Figure 2) has a 2,3-dihydro-2-phenylchromen-4-one structure. They are very abundant in citrus fruits and tomatoes. They seem to be more bioavailable than other close flavonoids such as flavonols or flavan-3-ols. This can be due to the fact that these compounds are less degraded by colonic microbiota and therefore they are more available for absorption [24]. The reason for this can be their common presence in food as rutinosides (bound to the disaccharide rutinose: 6-O-*α*-L-rhamnosyl-D-glucose) and neohesperidosides (bound to the disaccharide neohesperidose: 2-O-*α*-L-rhamnosyl-D-glucose), a tailoring that seems to be resistant to some colon microbiota species. In both cases, these disaccharides are attached at position C7. By contrast, flavanone glucosides are rare.

Flavanones deglycosylation and further degradation by colonic microbiota pathway is similar to that observed in flavonols (Figure 5), with the main difference being C-ring cleavage between C1 and C2 positions or between C4 and C10 ones.* Clostridium *species and* E. ramulus *are able to carry out these transformations in the colon [21, 24].

The flavanone hesperetin aglycon (e.g., from citrus fruits) shows a notable inhibitory activity against vancomycin-intermediate* Staphylococcus aureus* (VISA) and against* Helicobacter pylori *[75, 76]. It possesses also a synergistic effect on VISA when combined with antibiotics like vancomycin and oxacillin [75]. It inhibits also intracellular replication of diverse virus (herpes simplex virus type-1, poliovirus type-1, parainfluenza virus type-3, influenza A virus, and respiratory syncytial virus) [77, 78].

Its glycosylated flavanone, hesperidin, shows antibacterial activity against* Aeromonas hydrophila*, an emerging human pathogen that causes both intestinal and extraintestinal infections. In a murine model, hesperidin showed inhibition of bacterial colonization and a significant increase in anti-LPS IgM levels and reduction of anti-LPS and anti-ECP IgA levels to their normal values [79]. Hesperidin also shows activity against infection with human rotavirus [80] and against influenza virus replication* in vitro* by inhibition of the viral sialidase activity [81]. Growth of fungus* Phytophthora citrophthora* has been inhibited* in vitro* with this glycosylated flavanone, suggesting its role as antifungal toxin in the fruits of* Citrus sinensis, *a big source of this flavanone [82]. Also antiparasitic activity of hesperidin* in vitro* and* in vivo* has been shown against adult worms of* Schistosoma mansoni*, the causative agent of schistosomiasis [83].

Sulphonated hesperidin, one of its plasma metabolites, inhibits pathogens like* Chlamydia trachomatis* and* Neisseria gonorrhoeae in vitro* [84]. This conjugate also inhibits the enveloped viruses herpes simplex virus type-2 and human immunodeficiency virus (HIV) to the point that it has been suggested as a contraceptive antimicrobial agent against HIV transmission [84].

### 2.3. Flavan-3-Ols

Flavan-3-ols (Figure 2) form a very complex group of flavonoids consisting of simple flavan-3-ols (catechin and epicatechin; gallocatechin, epigallocatechin, and the corresponding gallate esters) and their polymeric forms. They are abundant in green tea, cocoa, kola, banana, and pomegranate.

Such broad polymerization degree and galloylation determine their bioavailability, as oligomers with a degree of polymerization >3 are not absorbed in the small intestine, and therefore they are metabolized in the colon [19, 25]. Their gallate esters are catabolised by colon microbiota, as, for example, epicatechin gallate and epigallocatechin gallate, generating aglycones and gallate, which is further decarboxylated into pyrogallol [25, 26, 85].

Flavan-3-ols aglycones lack a carbonyl group at C4 (as present in flavonols and flavanones). This may be the reason to avoid its transformation by colonic microbiota which modifies other types of flavonoids, as* E. ramulus *[27].

**Figure 6:**
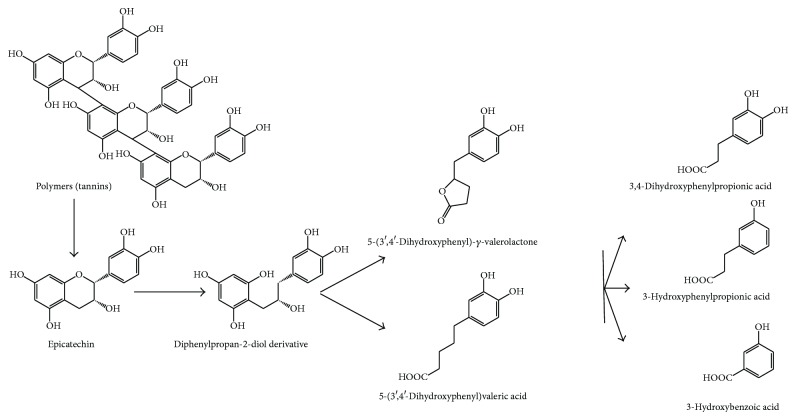
Colonic degradation of epicatechin tannins, as an example of flavan-3-ol polymers.

Once the initial gallate esters have been metabolized, the aglycones suffer C-ring opening, giving rise to diphenylpropan-2-diol, which is further converted into 5-(3′,4′-dihydroxyphenyl)-*γ*-valerolactone. This lactone ring opens and gives rise to 5-(3,4-dihydroxyphenyl)valeric acid. Further transformations generate OH-phenylpropionic and hydroxy-benzoic acids [25, 27] (Figure 6) (Table 1). Bacteria responsible for these metabolic reactions belong to the genera* Bifidobacterium *(as* Bifidobacterium infantis*) and* Clostridium *(as* Clostridium coccoides*). Actually, colonic populations of* Bifidobacterium* are increased in subjects consuming high doses of flavan-3-ols [28], which further enhance the benefits of flavan-3-ols consumption. These bacteria are resistant to these compounds because they do not use heme-containing enzymes, and these flavan-3-ols are important iron-chelating compounds [86].

In recent years, several studies have reported that the main catechin of green tea leaves, epigallocatechin-3-gallate (EGCG), has anti-infective properties [87]. Inhibition effect of EGCG on the capacity to infect cells by several viruses has been reported by different authors, who found that EGCG inhibits entry of hepatitis C virus by impairing virus binding to the cell surface [88–90]. EGCG also shows antiviral effects against HIV-1, interfering with several aspects of its life cycle. It interacts with the viral envelope destroying viral particles [91], prevents attachment of virions to cells downregulating CD4 cell surface receptor expression [92, 93], affects viral replication via inhibition of reverse transcription [94], and inhibits proviral genome integration by binding between the integrase and the viral DNA [95]. The antiviral activity of EGCG against influenza virus infection in cell culture was attributed to agglutination of virus particles thus preventing virus from adsorbing to cells [96]. EGCG also inhibits the acidification of endosomes and lysosomes required for the fusion of viral and cellular membranes [97] as well as of neuraminidase activity responsible for preventing self-aggregation of virus particles [98]. Clinical studies performed to investigate the preventive effect of catechins consumption on influenza infection in humans found this statistically significant [99, 100]. Enterovirus 71 [101], human hepatitis B virus [102], adenovirus [103], Epstein-Barr virus [104], and herpes simplex virus [105] are also clearly affected by EGCG.

With regard to antibacterial activity, there are multiple mechanisms by which EGCG exerts this activity against* Staphylococcus*, including damage to the lipid bilayer of the cell membrane [106], decrease slime production and inhibition of biofilm formation [107], binding and neutralization of enterotoxin B [108], and working with a synergistic effect in combination with *β*-lactams [109] or carbapenems [110]. Other bacteria killed by the action of EGCG are* Streptococcus pyogenes* [111],* Bacillus *spp. and* Clostridium *spp. [112],* Salmonella typhi* [113], and enterohemorrhagic* E. coli* [114]. The general antibacterial property of flavan-3-ols explaining these effects can be their chelating properties on iron, an important oligoelement for heme-utilizing bacteria [25]. EGCG inhibits growth of* Legionella pneumophila* inside macrophages not by any direct antibacterial effect on the pathogen, but due to selective changes in the immune response of macrophages and enhanced production of cytokines [115].

The antimicrobial effect of EGCG is also extended to eukaryote microorganisms, as against* Candida *spp. and the dermatophytes* Cryptococcus neoformans* and* Trichophyton mentagrophytes* [116]. EGCG specifically inhibits the germination of* T. mentagrophytes* conidia and subsequent hyphal growth [117]. These positive led to establishing* in vivo* research with EGCG in a murine model of disseminated candidiasis, showing its antifungal activity* in vivo *and its synergistic effect when combined with amphotericin B [118].

EGCG inhibits epimastigotes growth of* Trypanosoma cruzi* and increases mice survival rates in EGCG-treated animals that point out to a potential new compound for chemotherapy of Chagas disease [119]. EGCG also inhibits 37%–80% of binding of various isolates of* Plasmodium falciparum* to the ICAM-1 cellular receptor related to cerebral malaria [120]. The lethal mitochondrial damage that EGCG causes to* Leishmania donovani* [121] and* Leishmania amazonensis* [122] has been explained by its inhibition in the enzymatic activity of the parasite arginases [123].

### 2.4. Anthocyanidins

Unlike other flavonoids that are absorbed and secreted, anthocyanins, the glycosylated versions of anthocyanidin aglycons (as cyanidin, pelargonidin, and malvidin), do not appear to undergo extensive metabolism of the parent glycosides to glucuronic, sulfo or methyl derivatives, and therefore their bioavailability is very low [24]. Procyanidins occur in monomeric as well as in oligomeric and polymeric forms and are the most abundant and bioactive dietary polyphenols, as they are responsible for most red, blue, and purple color in fruits (specially berries), flowers, and leaves, besides having an important antioxidant activity [29, 30].

**Figure 7:**
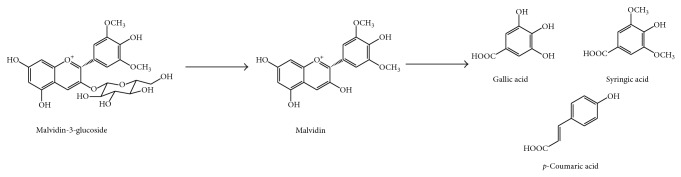
Colonic degradation of malvidin-3-glucoside, as an example of anthocyanin.

Since only a small part of ingested anthocyanins is absorbed at small intestine, large amounts of these compounds are likely to enter the colon, where they are deglycosylated by gut microbiota [124]. The gut microbiota has a high hydrolytic potential and ring scission properties so several anthocyanins degradation products have been identified. Some of them include vanillic, phloroglucinol, and protocatechuic acid [124, 125]. For example, incubation of malvidin-3-glucoside (from grape extracts) with fecal bacteria results in formation of gallic, syringic, and* p*-coumaric acids (Figure 7) (Table 1). Some species responsible for this degradation are* Lactobacillus plantarum*,* Lactobacillus casei*,* Lactobacillus acidophilus*, and* Bifidobacterium lactis *[29, 30]. All the anthocyanins and their metabolites tested significantly enhance growth of* Bifidobacterium *spp.,* Lactobacillus *spp., and* Enterococcus* spp. Therefore anthocyanins and their metabolites could perform a positive modulation of intestinal bacterial populations [126].

There are different mechanisms that can explain the antimicrobial activity of anthocyanins, as they can cause localized disintegration of bacterial outer membrane, leaking of cytoplasm (with the presence of significant amounts of cytoplasmic material and membrane debris outside the cells), and irregular shape [127]. The mechanisms thought to be responsible for the toxicity of pure anthocyanidin compounds to microorganisms include enzyme inhibition by the oxidized compounds, possibly through reaction with sulfhydryl groups or through more nonspecific interactions with proteins often leading to inactivation of the membrane protein and loss of function. Probable targets in the microbial cell are surface-exposed adhesions, cell wall polypeptides, and membrane-bound enzymes. Anthocyanidins may also render substrates unavailable to microorganisms, as some oligoelements [128, 129].

Many studies have shown the antimicrobial activities of the crude extract, fractions, and pure anthocyanidins from different berries. In bilberries (*Vaccinium myrtillus*), anthocyanins comprise 90% of the phenolic compounds. Extracts from bilberry and blueberry (*Vaccinium corymbosum*) showed inhibitory effects on the growth of Gram-positive bacteria (*Listeria monocytogenes*,* S. aureus*,* Bacillus subtilis*, and* Enterococcus faecalis*) and Gram-negative ones (*Citrobacter freundii*,* E. coli*,* Pseudomonas aeruginosa*, and* Salmonella enterica *ser*. Typhimurium*). However yeasts are resistant to these berry extracts [130].

Cyanidin-3-O-glucoside (C3G) inhibits the secretion of both VacA and CagA, two key virulence factors of* H. pylori* [131, 132]. C3G downregulates VacA secretion in* H. pylori* via inhibition of SecA expression (a protein involved in translocation of bacterial proteins out of the bacterial plasma membrane), causing a decrease in apoptosis in* H. pylori*-infected cells [132].

Cyanidin-3-sambubioside, a natural anthocyanin derived from black elderberry extract, binds to influenza virus neuraminidase within the 430-cavity, acting as a potent inhibitor of sialidase activity. This natural anthocyanin binds in the vicinity of neuraminidase residues 356–364 and 395–432, shielding proteases from releasing these peptide segments from the active site. This binding mode has not been seen with other influenza neuraminidase inhibitors so that the compound and its derivatives definitely offer the potential for the development of a new class of antivirals against influenza [133].

### 2.5. Isoflavones

Almost all isoflavones (daidzein, genistein, and formononetin) exist as glucosides and therefore are not absorbed across enterocytes due to their high polarity and molecular weight. These flavonoids are present almost exclusively in plants from the* Fabaceae* family (soy, lentils, beans, and chickpeas). Their bioavailability requires therefore conversion of glucosides into the bioactive aglycones via the action of intestinal *β*-glucosidases from small intestine bacteria (*Lactobacillus*,* Bifidobacterium*). Then, these aglycones are uptaken to the peripheral circulation [134].

**Figure 8:**
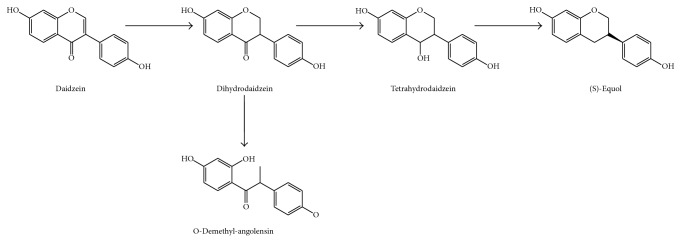
Colonic formation of (*S*)-equol and O-demethylangolensin from the isoflavone daidzein.

One of the most active isoflavones, daidzein, is metabolized in two different ways depending on subjects and their gut microbiota. Some subjects produce (*S*)-equol via dihydrodaidzein and tetrahydrodaidzein (resulting from the activities of* Streptococcus intermedius*,* B. ovatus*,* Ruminococcus productus*,* Lactobacillus mucosae* EPI2,* E. faecium *EPI1,* Veillonella *spp.,* Eggerthella* sp. Julong732, and* Finegoldia magna *EPI3) [31–33] (Figure 8). However others produce O-desmethylangolensin (O-DMA) via 2′-dehydro-O-demethylangolensin (generated by* Clostridium *spp.) [34] (Figure 8) (Table 1). Therefore, there are two groups of subjects, (*S*)-equol producers and nonproducers. The inability to produce (*S*)-equol is a consequence of the lack of specific components in the intestinal microbiota, as the species described before. (*S*)-equol shows high antioxidant activity due to its nonplanar structure, which enables it to penetrate more easily into the interior of the cell membrane, preventing oxidative damage* in situ*. Also its estrogenic activity on mammal cells is higher in comparison with other phytoestrogens. This compound binds to estrogen receptor in mammal cells, downregulating its activity. This may have potential application in breast and prostate cancer therapy and prevention [135–137]. In addition to (*S*)-equol and O-DMA, other less active microbial metabolites of daidzein have been reported [138].

Microbial metabolism of isoflavone genistein is different from that of daidzein. Genistein is reduced to dihydrogenistein, which is further metabolized to 6′-hydroxy-O-desmethylangolensin [35].

Other less common isoflavones found in red clover are formononetin and biochanin A, which are converted in a similar way to microbial metabolites. Formononetin is rapidly converted via daidzein to O-DMA and (*S*)-equol. Biochanin A is metabolized via genistein to 6′-hydroxy-O-desmethylangolensin [36]. Then, all these isoflavone aglycones are further transformed by C-ring cleavage and dehydroxylation reactions in the colon.

Apart from their estrogenic activity, studies with respect to the antimicrobial activity of isoflavones have been described, as, for example, inhibition of* S. aureus* MRSA strains at concentrations over 128 *μ*g/mL [139–141]. These activities are thought to be due to inhibition of bacterial topoisomerase IV [142].

### 2.6. Flavones

These flavonoids (luteolin, apigenin) share the 2-phenylchromen-4-one (2-phenyl-1-benzopyran-4-one) backbone. They are present in food as cereals, parsley, thyme, celery, and citrus fruits. Once the corresponding glucosides have been hydrolyzed at intestinal level, unabsorbed aglycons are further metabolized by colon microbiota (*C. orbiscindens*,* Enterococcus avium*), breaking down their C-ring towards phloretin chalcone, 3-(3,4-dihydroxyphenyl)-propionic acid, 3-(4-hydroxyphenyl)-propionic acid, 3-(3-hydroxyphenyl)-propionic acid, and 4-hydroxycinnamic acid, which are absorbed and excreted by urine [37] (Table 1).

Luteolin and its glycosides have been isolated from plants used in traditional medicine to treat a wide range of diseases. Tests for antiherpetic substances from crude methanol leaf extract of* Avicenna marina* have shown that the most active fraction isolated and analyzed contained luteolin 7-O-methylether-3′-O-beta-D-glucoside (LMEG). LMEG exerts an inhibitory effect on the early stage of herpes simplex virus 2 (HSV-2) infection probably inhibiting HSV attachment to the cell membrane and its entry into the cell [143]. Among several compounds isolated from* Swertia macrosperma*, luteolin was the most active compound in inhibiting the secretion of hepatitis B virus surface antigen (HBsAg) and hepatitis B virus e-antigen (HBeAg) with IC50 values of 0.02 and 0.02 mM, respectively [144].

Regarding the antibacterial effects, luteolin is active against* B. subtilis*,* S. aureus*,* P. fluorescens* and* E. coli* [145, 146]. The major constituents isolated from the methanol extract of* Daucus carota *(carrot) seeds are luteolin, luteolin-3′-O-beta-D-glucopyranoside, and luteolin-4′-O-beta-D-glucopyranoside. Both luteolin and its 4′-O-glucoside demonstrated bactericidal activity against* S. aureus* and* E. coli* (MIC = 5.0 × 10^−2^ and 1.0 × 10^−1^ mg/mL, resp.) [147]. Luteolin shows antibacterial and synergistic activity against amoxicillin-resistant* E. coli*, acting via three mechanisms: inhibition of proteins and peptidoglycan synthesis, inhibition of extended-spectrum *β*-lactamases, and alteration of outer and inner membrane permeability [148].

Luteolin and its glycosides also show antiparasitic activity. Luteolin present in extract from* Melampyrum arvense* was the most active compound against* Trypanosoma brucei *ssp.* rhodesiense* and* L. donovani* (IC(50) values 3.8 and 3.0 *μ*g/mL) [149]. Luteolin-7-O-*β*-glucopyranoside displayed the best antiplasmodial activity against* P. falciparum* (IC(50) value 2.9 *μ*g/mL) [149].

### 2.7. Hydrolyzable Tannins

Hydrolyzable tannins are a class of polyphenols that include gallotannins and ellagitannins (ETs) (Figure 4). These compounds are present in fruits like raspberry, cranberries, strawberries, walnuts, grapes, and pomegranate. A main difference between these two groups is that, upon gut microbial hydrolysis, gallotannins yield glucose and gallic acid, whereas ellagitannins undergo lactonization producing ellagic acid (Figure 4).

Ellagic acid is largely metabolized by the colon microbiota, giving rise to urolithin A (3,8-dihydroxy-6H-dibenzopyran-6-one) and its monohydroxylated analog known as urolithin B [150, 151]. There is a large interindividual variation in the timing, quantity, and types of urolithins excreted in urine by humans. These variations are due to the variations in colonic microbiota composition [152, 153]. Despite all the data indicating the microbial origin of urolithins, no specific bacteria for urolithin biosynthesis have been yet identified. One bacterium (*Butyrivibrio* spp.), responsible for ellagitannins modification, has been identified in rumen fluids [38] (Table 2).

Ellagic acid from* Phyllanthus urinaria*, a domestic plant grown in Korea, shows specific antiviral activity against hepatitis B virus (HBV), by inhibiting HBeAg secretion, in HBV-infected cells [154]. ETs are potent antiviral agents against herpes simplex virus, specially eugenin extracted from* Geum japonicum* and* Syzygium aromaticum *[155]. Pomegranate (*Punica granatum*) polyphenols suppress the replicative ability of influenza A virus in host cells. Punicalagin is the most effective anti-influenza component in this extract, blocking replication of influenza virus RNA and inhibiting agglutination of chicken red blood cells by the virus [156, 157]. Geranin and corilagin are two ETs extracted from* Phyllanthus amarus* restrained by 50% the interaction of glycoprotein 120 of HIV-1 at concentrations from 2.65 to 0.48 *μ*g/mL on the primary cellular receptor CD4 [158].

Plant extracts from* Pteleopsis hylodendron,* containing mainly ellagic acid, are active against* Klebsiella pneumoniae*,* Bacillus cereus*,* E. coli*, and* S. typhi* [159]. ETs present in pomegranate peel are effective also in inhibiting* S. aureus*,* Salmonella*,* L. monocytogenes*, and* E. coli* [160–164]. Ellagic acid extract from pomegranate inhibits formation of biofilms by* S. aureus*, methicillin resistant* S. aureus* (MRSA), and* E. coli*. [79].

Punicalagin, punicalin, gallagic, and ellagic acids show antifungal properties against* Candida albicans*,* C. neoformans*, and* Aspergillus fumigatus* [165]. Apart from inhibiting biofilm formation, pomegranate extracts disrupt preformed biofilms and inhibited germ tube formation in* C. albicans* [164].

*In vitro* antimalarial activity of ellagic acid has been reported, with high* in vitro *activity against all* P. falciparum* strains regardless of their levels of chloroquine and mefloquine resistance (50% inhibitory concentrations ranging from 105 to 330 nM) [166]. This antimalarial activity takes place at the mature trophozoite and young schizont stages, corresponding to protein and nucleic acid synthesis. Ellagic acid potentiates also the activity of current antimalarial drugs such as chloroquine, mefloquine, artesunate, and atovaquone [167].

### 2.8. Lignans

Lignans include a number of diphenolic compounds with a 1,4-diarylbutane structure such as secoisolariciresinol, matairesinol, pinoresinol, lariciresinol, isolariciresinol, and syringaresinol. They are common in seeds as flax and cereals and in fruits as strawberries and apricots.

**Figure 9:**
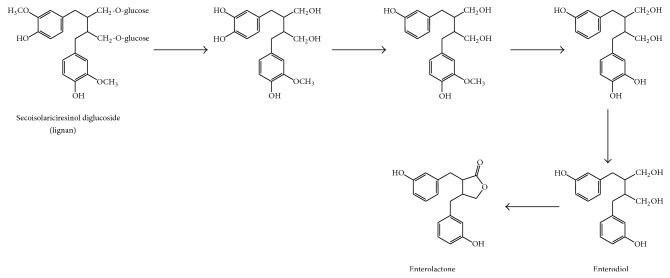
Colonic formation of enterodiol and enterolactone from the lignan secoisolariciresinol diglucoside.

Lignan metabolism involves both mammalian (glucuronidation and to a lesser degree sulfation) and gut microbial processes [39]. Biological activity of lignans is related to the activation of these compounds by* Bacteroides and Clostridium* species (in the gut microbiota) to enterolactone and enterodiol (Figure 9), which are phytoestrogens in mammals [40]. This transformation of lignans into phytoestrogens is carried out after demethylation and dehydroxylation reactions (carried out by* Peptostreptococcus *and* Eubacterium *species), which increase the structural diversity of enterolignan derivatives in blood circulation (Table 2) [41] (Figure 9). Thus, enterolactone conversion from enterodiol is a complex phenomenon, involving several precursors, different intermediary metabolites, and diverse conjugation patterns. Production of enterolactone was compared to that of enterodiol and a ratio of enterolactone- and enterodiol-converting bacteria of 1 : 2000 was observed, indicating that enterodiol-producing bacteria are dominant in human gut [42] (Table 2).

### 2.9. Chlorogenic Acids

Chlorogenic acids are a group of compounds comprising hydroxycinnamates (such as caffeic acid, ferulic acid, and* p*-coumaric acid) (Figure 1), linked to a quinic acid to form a range of conjugated structures known, respectively, as caffeoylquinic acids, feruloylquinic acids, and* p*-coumaroylquinic acids. They are abundant in fruits as peaches and plums and in some seeds, like coffee.

Literature describing the bioavailability of chlorogenic acids is scarce and contradictory. However, several microbial metabolites have been identified. The main microbial metabolites of caffeic acid are 3-hydroxyphenylpropionic acid and benzoic acid, generated by the action of* E. coli*,* B. lactis*, and* Lactobacillus gasseri* (Table 2). The first one is formed by de-esterification, reduction of a double bond, and dehydroxylation. Furthermore, *β*-oxidation shortens the side-chain and forms benzoic acid in small degree. Both metabolites are also obtained from chlorogenic acid [43]. The most frequent metabolites from ferulic acid produced by colonic microbiota are vanillin and 3-(4-hydroxyphenyl)-propionic acid [44, 45].

The antimicrobial activity of 22 polyphenols, including gallic acid, was investigated against 26 bacterial species. It was found that a structure-activity relationship between the strongest antibacterial activity for those polyphenols and a higher number of pyrogallol rings in their structure [168]. As gallic acid has one of those rings, its antibacterial activity was classified by these authors as moderate. The role of gallic acid is also of practical interest in the prevention of formation of biofilms by different bacteria. When biofilms formed by* E. coli*,* P. aeruginosa*,* S. aureus*, and* L. monocytogenes* were studied, a reduction in biofilm activity >70% for all the biofilms tested was found [169]. Gallic acid also inhibits bacterial growth of* Streptococcus* mutants and the biofilm formation* in vitro* and also influences the adhesion properties of* S. aureus* [170].

Experimental evidences regarding the antiviral activity of gallic acid have been published as the inhibition in human rhinoviruses (HRVs), replication and reduction of HRV-induced cytopathic effect* in vitro*, and antienterovirus 71 activity [171] were found. The same positive results of gallic acid against herpes simplex virus type-2 were previously mentioned [172].

Gallic acid purified from* Terminalia nigrovenulosa* bark has shown strong antifungal activity against* Fusarium solani* and strong nematicidal activity against* Meloidogyne incognita *[173, 174].

## 3. Conclusions

Most polyphenol nutraceuticals from plant origin must undergo intestinal transformations, by microbiota and enterocyte enzymes, in order to be absorbed at enterocyte and colonocyte levels. This gives rise to diverse beneficial effects in the consumer, including a vast array of protective effects against viruses, bacteria, and protozoan parasites. These enzymatic transformations include elimination of glycosidic tailoring by gut microbiota of diverse genera (*Lactobacillus*,* Eubacterium*, and* Bifidobacterium*), as well as further transformations in these aglycones' level, giving rise to more stable bioactive compounds that are incorporated into the blood stream, as a vast array of benzoic acids, phenolic acids, urolithins, and the phytoestrogens (*S*)-equol, enterodiol, and enterolactone. In most cases, a complex network of different intestinal microbiota species is necessary for full biotransformation, whereas earlier and simple reactions as deglycosylation can be carried out individually by specific gut strains. The individual variability, at consumer level, with respect to richness, and biodiversity of own intestinal microbiota taxa are key determinants regarding the ability of a person to get the most fully bioactive derivatives from ingested polyphenols. Final absorbed bioactive derivatives have shown antimicrobial properties against viruses (as HBV), Gram-positive bacteria (as* S. aureus*,* L. monocytogenes)*, and Gram-negative bacteria* (S. enterica*,* P. aeruginosa*), but also against eukaryote species as fungi (*Candida *spp.,* T. mentagrophytes*) or protozoans (*T. cruzi*,* P. falciparum*). Therefore, consumption of food with high levels of polyphenols, together with having appropriate gut microbiota diversity, is extremely important, in order to help in the fight against infectious diseases. Fermented dairy foods, as well as other ones with high levels of beneficial microorganisms, can therefore contribute to maintaining this appropriate gut microbiota diversity, facilitating intestinal production of bioactive metabolites from dietary polyphenols, as well as their absorption and bioavailability.

## Figures and Tables

**Table 1:** Main metabolites derived from flavonoids and identified bacteria involved in their transformation.

Precursors		Main metabolites identified	Bacteria	References
	Kaempferol	2-(4-Hydroxyphenyl)propionic acid	*Clostridium orbiscidens *	[20]
Flavonols	Quercetin	2-(3,4-Dihydroxyphenyl)acetic acid 2-(3-Hydroxyphenyl)acetic acid 3-(3,4-Dihydroxyphenyl)propionic acid 3-(3-Hydroxyphenyl)propionic acid	*C. orbiscidens, Eubacterium oxidoreducens* *Eubacterium ramulus* *Enterococcus casseliflavus *	[21–23]
	Myricetin	2-(3,5-Dihydroxyphenyl)acetic acid 2-(3-Hydroxyphenyl)acetic acid	*C. orbiscidens, E. oxidoreducens *	[20, 22, 23]

Flavanones	Naringenin	3-(4-Hydroxyphenyl)propionic acid	*Clostridium* strains *E. ramulus *	[21, 24]

Flavan-3-ols	Catechin Epicatechin	3-(3-Hydroxyphenyl)propionic acid 5-(3′,4′-Dihydroxyphenyl)-*γ*-valerolactone 5-(3,4-Dihydroxyphenyl)valeric acid 3-(3,4-Dihydroxyphenyl)propionic acid	*Clostridium coccoides, Bifidobacterium *spp.	[25–28]
	Epigallocatechin	5-(3′,4′-Dihydroxyphenyl)-*γ*-valerolactone 5-(3′,5′-Dihydroxyphenyl)-*γ*-valerolactone		

Anthocyanins	Cyanidin	3,4-Dihydroxybenzoic acid	*Lactobacillus plantarum, Lactobacillus casei, Lactobacillus acidophilus* LA-5, *Bifidobacterium lactis* BB-12	[29, 30]
	Peonidin	3-Methoxy4-hydroxybenzoic acid		
	Pelargonidin	4-Hydroxybenzoic acid		
	Malvidin	3,4-Dimethoxybenzoic acid		

Isoflavones	Daidzein	(*S*)-Equol	*Bacteroides ovatus*, *Streptococcus intermedius*, *Ruminococcus productus*, *Eggerthella *sp.Julong 732, *Enterococcus faecium* EPI1, *Lactobacillus mucosae* EPI2, *Finegoldia magna *EPI3	[31–33]
		O-Demethylangolensin	*Clostridium *spp. HGHA136	[34]
	Genistein	6′-Hydroxy-*O*-desmethylangolensin		[35]
	Formononetin	Daidzein		[36]
	Biochanin A	Genistein		[36]

Flavones	Luteolin, apigenin	3-(3,4-Dihydroxyphenyl)-propionic acid, 3-(4-hydroxyphenyl)-propionic acid, 3-(3-hydroxyphenyl)-propionic acid, and 4-hydroxycinnamic acid, phloretin	*C. orbiscindens, Enterococcus avium *	[37]

**Table 2:** Main metabolites derived from nonflavonoids and identified bacteria involved in their transformation.

Precursors	Main identified metabolites	Bacteria	References
Ellagitannins	Urolithins	*Butyrivibrio* spp.	[38]

Lignans	Enterodiol Enterolactone	*Bacteroides distasonis*, *Bacteroides fragilis*, *Bacteroides ovatus, Clostridium cocleatum, Butyribacterium methylotrophicum, Eubacterium callanderi, Eubacterium limosum, Peptostreptococcus productus, Clostridium scindens, Eggerthella lenta *	[39–42]

Hydroxycinnamates	3-Hydroxyphenylpropionic acid Benzoic acid 3-(4-Hydroxyphenyl)propionic acid Vanillin	*Escherichia coli, Bifidobacterium lactis, Lactobacillus gasseri *	[43–45]
